# Alcohol Use, Risk Taking, Leisure Activities and Health Care Use Among Young People in Northern Vietnam

**DOI:** 10.5195/cajgh.2013.10

**Published:** 2013-10-01

**Authors:** Le Thi Kim Thoa, Dang H. Hoang, Nguyen Dang Vung, Pham H. Tien, M.A. Plant

**Affiliations:** 1 Ha Noi Medical University, Vietnam; 2 Center for Health and Development in Vietnam; 3 Alcohol & Health Research Unit, University of the West of England, Bristol, United Kingdom

**Keywords:** Alcohol, young people, Vietnam, risk taking, leisure, health care

## Abstract

Alcohol consumption is associated with a wide range of health and social consequences. It is also associated with a number of risk taking behaviours. These include illicit drug use and unsafe sex. Alcohol consumption appears to be increasing in Vietnam. The purpose of this paper is to examine the patterns of alcohol consumption and its relationship with a number of other risk taking behaviours amongst young people. Information was also obtained concerning leisure activities and use of health care. The paper also sets out to examine possible gender differences in relation to alcohol consumption and risk behaviour and to propose the development and implementation of alcohol monitoring and prevention programs in Vietnam. The study involved a cross-sectional, community survey using a standardised interview. This was conducted during face-to-face interviews with 1,408 young people aged 10–19 years. Respondents were recruited randomly through the lists of the households from 12 selected communes in three areas in Northern Vietnam. The findings presented here were part of a larger health risk behaviour survey. Levels of alcohol use were low. Overall, 16.5% of participants were experienced drinkers, and only 4% of them were current drinkers. Males were significantly more likely than females to report drinking. This study also showed that rates of alcohol consumption were associated with age, education, geographical area, gender, tobacco smoking, involvement in violence, watching television, computer use and playing computer games, wearing safety helmets and use of health services. Alcohol consumption tended to increase with age for both males and females. Alcohol and its effects on young people are clearly a growing public health issue in Vietnam. Because of this, more detailed behavioral research should be conducted into the relationship between alcohol consumption and other risky behaviours amongst young people. It is also recommended that alcohol harm reduction policies should be implemented and integrated into measures to reduce levels of other health problems such as HIV/AIDS and non communicable diseases. Such policies should ideally be evidence-based and evaluated.

## Introduction

Alcohol consumption and tobacco smoking are associated with cardiovascular diseases, cancers, chronic obstructive pulmonary diseases and diabetes.^1^–^7^ Alcohol consumption is associated with “disinhibition” and possibly with sexually transmitted diseases, including HIV/AIDS, and the hazardous and harmful use of alcohol has now become one of the most important risks to health: it is the leading risk factor in developing countries with low mortality rates and ranks third in developed countries (according to the World Health report 2002). Evidence suggests that heavy drinkers may be inclined to take risks, regardless of whether or not they drink on a particular occasion.^8^,^9^ Moreover, the possible connection between drinking and sexual behaviour is at least partly attributable to the fact that many people meet their sexual partners in bars and other places where alcohol is consumed socially.^10^,^11^ Alcohol consumption, often involving home made drinks, is commonplace in Vietnam.^12^ Drinking is widespread amongst Vietnamese young people whose health has been considered a low priority.^13^ Heavy drinking by young people is associated with health and social problems, tobacco and illicit drug use and a range of other risky behaviours.^8^,^14^ Furthermore, when drinking and smoking are adopted by younger children, this behaviour appears to enhance risks of later alcohol and drug dependence, obesity, high blood pressure and other illnesses and problems.^15^

In the USA and elsewhere there has been an increasing body of literature that suggests a strong link between alcohol consumption and engagement in sexual risk behaviours.^16^ Analysis of Youth Health Risk Behavior data indicates that the youth who use alcohol are seven times more likely to be sexually active than the youth who do not drink alcohol, a figure higher than that for young people using illicit substances. Another recent report indicates that among sexually active high school students 39 percent of those consuming alcohol regularly have had sex with four or more partners, compared to 29 percent of non-drinkers.^16^ In addition a U.S. national survey indicated that almost 25 percent of sexually active young people and young adults (15 to 24 years) have engaged in unprotected sex while using alcohol or drugs. One survey showed that respondents who drank alcohol were seven times more likely to have sex as respondents who did not drink. Finally, a study of college students found that 40.7 percent of males and 27.8 percent of females stated that they had engaged in sexual intercourse under the influence of drugs and/or alcohol when they would not otherwise have engaged in these behaviours.^18^

Socio-cultural and demographic studies of alcohol use and abuse patterns have been conducted in Asia, including China and Thailand. Recent research in Asia also includes studies that examine alcohol consumption among young people. Among these, a study in Japan reveals that the youth are beginning to drink at a younger age, and rates of drinking have been on the rise among 13 to 17 year olds.^19^ Among South Korean young people, a fairly high rate of alcohol consumption has been demonstrated, with one study showing 43 percent of young people drinking regularly, and that boys are more likely to drink than girls.^20^ In addition, among a sample of 1,040 young people in grades 6, 8, and 10 in Beijing, China, approximately 70 percent have reported prior alcohol consumption. Again, males were significantly more likely to drink alcohol than females. Even so, 61% of females reported prior use of alcohol. Another study among senior high school students in China reported that 83.5 percent of boys and 54.9 percent of girls had consumed alcohol.^21^ There is, however, there is significantly less information available for Southeast Asia. Studies in Thailand have linked paternal drinking with subsequent alcohol use by their adult children and other studies examined links between alcohol consumption and HIV risk behaviors.^22^

“Official figures” indicate that per capita alcohol consumption in Vietnam is very low (0.9 litres of alcohol). This compares with 1.5 litres for Cambodia, 5.6 litres for Thailand and 7.6 litres for Japan.^23^ These figures do not include unrecorded alcohol, including home made drinks. However, it is evident that there has been an increase in alcohol consumption. The World Health Organization has noted that the recorded alcohol consumption in Vietnam rose from approximately 0.7 litres in 1961 to more than 1.3 litres in 2001.^24^ It further estimated that the unrecorded alcohol consumed amounted to one litre of alcohol per head of those older than 15 years. The World Health Organization also reported that 69.5% of Vietnamese adults were lifetime abstainers, 2.9% were heavy and hazardous drinkers, and 4.7% were heavy episodic (or binge) drinkers. Moreover, it reported that 80% of those aged 18–24 years were drinkers, of whom 3.7% were heavy episodic drinkers.

Evidence suggests that as countries develop economically, there is an increase in alcohol consumption.^25^ For example, in Thailand, the rate of alcohol consumption increased 334 percent from 1.93 litres in the early 1970s to 8.64 litres in 1996. With limited socio-religious restrictions in Vietnam on drinking, one could anticipate that alcohol consumption will continually increase as economic conditions improve. There has, however, been little research in Vietnam on alcohol use within the adolescent population.

This paper examines alcohol consumption and its association with gender in relation to a number of risky behaviours and use of health services amongst young people in Vietnam. The paper also seeks to examine possible gender differences in relation to these variables.

## Methods

This study involved a cross-sectional population survey of young people who were identified from the updated list of all households at commune level. This list was used to select a stratified sample of addresses. Fieldwork (including pilot work) took place between June 1st and August 30th, 2004 in Ha Noi City, Ha Nam and Thai Nguyen province, Vietnam.

### Participants

The participants were young people in twelve communes of three sites. Young people were between 10 and 19 years of age. They agreed to participate in the survey. Fieldworkers conducted short interviews with parents before conducting interviews with the young people to get some family information including some details of parental drinking and smoking. Parents were not present during interviews with their children. The response rate for parents was high; no one refused to take part. All interviews were carried out in private.

### Sample design and sampling

The sample design utilised in this study is shown in Table 1. It was a multi-stage stratified sample that was representative of young people aged 10 to 19 years. In the first stage, three sites (one city and two provinces), were selected from 64 provinces and cities in Vietnam. These sites represent three geographically well-defined areas in the North. Ha Noi City is in the urban area located in the Red River Delta and is also the Capital of Vietnam with 3,082,800 inhabitants (in 2004). Ha Nam Province is in the rural area with its population of 820,100 (in 2004) and Thai Nguyen Province is in the mountainous midland area with a population of 1,095,400 (also in 2004). Twelve commune units (SCUs) were selected during the second sampling stage. The population of each SCU ranged from 5,000 to 10,000. In the final stage of the design, all households which included young people in the target age group in these selected communes were eligible to participate in the survey. The list of all households with such young people was provided by health workers working in local health centers. All the young people were randomly selected for interview, resulting in the sample size of 1,442 individuals.

All individuals aged from 10 to 19 randomly sampled were single and available at home during the survey. (Most people in Vietnam marry after the age of 20, except some cases in rural and mountainous areas). The participants were randomly selected by Stata software program. Out of a total of 1,440 people who were contacted, 1,408 young people (97.8%) participated in the survey. A total of 32 individuals (2.2%) declined to participate. The data from these 12 SCUs applied only to young people participating in the survey.

### Research procedure

Before conducting this survey, the investigators conducted a qualitative research (individual interview and focus groups) as a pilot study. They tested both the questionnaire and survey procedure with boys and girls from the general population.

### Questionnaire development

The questionnaire was developed in three stages. The first stage involved a qualitative research including 30 individual interviews, using both the unstructured interview guidelines and the semi-structured interviews form. These interviews were conducted to explore the perceptions of young people about healthy and risky behaviors. They also examined perceptions of risk practices and solutions to prevent such behaviours among young people. The information obtained from these interviews identified health behaviours and other related factors. The most commonly reported seven risk behaviours perceived by young people included drug use, fighting, motorcycle racing, alcohol consumption, tobacco smoking, lack of exercise or sporting activities, eating snacks in the street, driving bicycle or motorcycle without a helmet and unprotected sex. The second stage involved three focus group discussions (FGDs) with both male and female young people. These groups each included between ten and eighty participants. Participants were asked for advice about the form and content of questionnaire. Those taking part in FGDs suggested that the instrument should be divided into two versions. The first version should be used for all participants (10–19 years old). The second version (including items about HIV/AIDS knowledge and sexual practices) should be used for participants from 15 or older. The participants in FGDs reported that the questions in version 2 would be too sensitive for very young people. In the last stage, the adolescent health risk behavior survey (AHRBS) components of the questionnaire included five main components: 1) demographic information; 2) alcohol consumption details; 3) tobacco use; 4) Watching television, using a computer or playing computer games; 5) violence associated with drinking; 6) riding bicycle and motorbicycle and helmet use; 7) use of medical services.

### Data collection and analysis

Participation in the study was informed and voluntary. Data collection was anonymous. The surveys were conducted by health staff using face-to-face interviews carried out in respondents’ homes. Respondents typically completed interviews in one hour. Their responses were recorded directly onto the questionnaire. The core questionnaire contained 65 multiple-choice questions. Before the survey was administered, local consent procedures were followed. Collaborating with the city/provincial Bureau of Health, the investigators selected the communes as well as certain admission criteria. The administrators at each selected locality first asked permission from the Ha Noi Medical University and the local health administration. This specific study was approved by the Scientific and Ethical Committee for Medical Research, Hanoi Medical University and the local authorities. Before the interviews were conducted, local parental permission procedures were followed. For surveying participants younger than 18 years of age their parents agreed for their children to participate in advance. Informed consent was also were obtained from all respondents before interviews were conducted.

The data entered into the EpiInfo 6.04. SPSS, version 15.0 for windows for analyzing frequencies, proportions (percentages), odds ratio (OR) and using χ2 test with 95% confident interval (CI). Multiple regression analyses were estimated for the relationship between alcohol use and other variables.

## Results

### Characteristics

As noted above, information was elicited from 1,408 young people. A total of 673 males (48%) and 735 females (52%) were included in the sample obtained. All these young people were unmarried. The mean age of the sample was 14.35 years (SD = 2.6) years. Mean age was 14.27 years (SD = 2.6) years for males and 14.43 years (SD = 2.6) for females. Most young people were the Kinh (94%). Other ethnic minorities (4%) included Tay, Nung and San Diu groups. Most of these ethnic groups lived in the mountainous areas. No difference was found in age and gender between the two groups (χ2: 1.04, d.f. = 2, p = 0.307). Approximately 94% of the young people reported that they were attending school at the time of the survey. Among those who were not in school (86) the number was the lowest in urban (2.6%) areas and higher in rural areas (10.7%). No difference in school attendance was evident between the two gender groups (χ2: 0.48, d.f. = 2, p = 0.488). There were no statistically significant differences in gender (p > 0.1), age, education levels and georgraphical study sites (see Table 2).

### Self report alcohol use

In general, most of young people surveyed, 83.5%, reported that they had never consumed alcohol. The proportion of female non-alcohol users was higher than males (89.4% vs 77%). Overall, 16.5% (233) of the sample reported they had consumed alcohol. A total of 23% of males reported drinking more than females (10.6%) (OR= 2.52; 95% CI, 1.86 – 3.42; p = 0.001). Alcohol consumption was significantly higher amongst of older males (37.1%) than among younger males (12%) (OR = 4.98, 95% CI, 3.25 – 7.63; p = 0.000). This age difference was also evident amongst females (7.8%) (OR = 6.97, 95% CI, 4.38 – 11.13; p = 0.000). Young people who were not attending school (24.6%; n = 17/69) were significantly more likely to drink alcohol than were in-school respondents (OR = 1.26; 95% CI, 0.70 – 2.25, p = 0.01). Among the 233 drinkers, 97% (n = 226), reported that they had never consumed alcohol daily. Only five boys and two girls reported they had ever drunk on a daily basis. A significantly greater proportion of young drinkers came from the mountainous areas, compared to those from the rural and urban areas (χ2 =15.55, d.f. = 2, p = 0.0004). The proportion of young people reporting alcohol use was higher among out of school respondents (24.6%; n = 17/69) compared to young people who were at school (OR = 1.26; 95% CI, 0.70 – 2.25, p = 0.01). Alcohol use tended to increase by age (10–11 age: 6.3%; 12–13 age: 11.3%; 14–15 age: 11.0%; 16–17 age: 27.8%; and 18–19 age: 29.5%), and also with education level (primary: 7.6%; secondary: 12.5%; high: 28%; University: 35.7%). There was a significant different amongst males in the 10–11 age group (6.8%) and the 18–19 age group (53%) (OR = 15.46; 95% CI, 6.58 – 37.32, p = 0.000). This difference was also evident by gender in most subpopulations (see Table 3).

### Age of First Frequent Drinking

Respondents were asked when they had “first started drinking frequently.” Most (77%) of those who had consumed alcohol reported that they had “never drunk regularly.” The proportion of the females (84%) who replied in this manner (“never drunk regularly”) was higher than that of males (74%). Amongst drinkers, 23% (52) reported drinking as frequently as once a month or more. For those under 14 years of age, drinking regularly was not a common practice. However, among the 233 drinkers, four males and three females reported having begun to drink frequently at “10 years of age or younger.” Five of these individuals had started to drink frequently at 10 to 14 years of age. Two young people reported that they started to drink at “15 to 19 years of age” and 43 did not answer (the response rate for this question was 81.2%).

### Current Drinking

Current drinking was reported by only 55 (3.9%) of the young people who were surveyed. Amongst this small group two young people (one male and one female) were drinking daily.

### Past week’s alcohol consumption

Respondents were asked to provide details of any alcohol they had consumed in the past week. Most (82.8%) reported that they did not drink at all (82.8%) and only 40 (2.8%) reported that they had consumed alcohol during the past week. The latter included 33 males and 7 females. The proportion of male young people reported on drinking alcohol in the past seven days was four times higher than females, but three-quarters of them only drank once. Among drinkers, the percentage of those who reported drinking twice or more during the past seven days was 4.3%.

### Factors associated with alcohol consumption

#### Tobacco smoking

Overall, 37 (2.6%) respondents had smoked using filter cigarettes. None of the females had ever smoked. The proportion of smokers was higher among older boys (11.2%) compared to younger boys (0.5%) (OR = 22.25; 95% CI: 5.17–134.92; p = 0.000). Drinkers were significantly more likely to smoke than non-drinkers (OR = 9.09; 95% CI: 4.26–20.61; p = 0.000). There was a strong association between alcohol use and smoking in both older and younger boys (OR = 10.05; 95% CI: 1.37–206.44; p = 0.002). The majority, 71.4% (n = 25/35), of smokers also reported having consumed alcohol (OR = 5.11; 95% CI: .23 – 11.94; p = 0.000).

#### Television and computer use

Ninety per cent of the young people who were surveyed reported that they watched television, used computers or played computer games on a daily basis. A total of 502 respondents, (39.6%), reported spending at least three hours each day on these activities. The amount of time spent on these activities did not differ by gender (see Table 4).

#### Alcohol-related violence

In all three study sites, 24.1% (340) of young people were involved in physical fighting at least once during the previous year. Males were significantly more likely (29.4%) than females to report such experience (19.3%) (OR = 1.74; 95% CI: 1.35–2.27; p = 0.000). Fighting amongst younger male drinkers was significantly more likely than younger male non-drinkers (OR = 1.51; 95% CI: 0.89–2.58; p = 0.106). Consistent with this, fighting was also more common place amongst older male drinkers than amongst older males who did not drink (OR = 140; 95% CI: 0.78–2.50; p = 0.222). Fighting among female drinkers was also significantly more likely than amongst females who did not drink alcohol (OR = 1.29; 95% CI: 0.71–2.33; p = 0.374).

#### Use of helmets by bicycle and motorcycle riders

The proportion of young people using bicycles and motorcycles was very high (86%; n = 1210) in all study sites. The riders included 572 males (85%) and 665 females (70%). The practice of wearing helmets was very low (n = 268; 22.1%). Overall only 27.3% of respondents reported that they ‘always’ wore helmets. Those young people who wore helmets at least sometimes (13.3%) were significantly more likely than those who ‘never’ wore helmets (3.6%) to report alcohol use (χ2 =45.31, d.f. = 2, p = 0.000). These findings are elaborated in Table 5. There was a significant difference for alcohol use difference by gender for all helmet users compared with non helmet wearers.

#### Medical check up

The proportion of young people who had received a medical check up one or more times in the past year was 56.6%. The proportion of those receiving such check ups was not significantly associated with gender (OR = 1.14; 95% CI: 0.92 – 1.42; p = 0.221). Even so, the probability of having had a medical examination was significantly associated with alcohol consumption. There was a strong association between alcohol use and frequency of medical check up in the past year (OR = 1.80; 95% CI: 1.34 – 2.42; p = 0.000). A disproportionate number, 110 out of 123 (89%) of those who had not received medical check ups also reported drinking on two occasions or more per year (OR = 1.77; 95% CI: 1.32 – 2.37; p = 0.000). Overall, young people who had not had medical check ups were significantly more likely than non-drinkers to be recent drinkers (OR =1.77, 95% CI: 1.32 – 2.37; p = 0.000). Drinkers appeared to care less for their health, so they had fewer regular check ups. Routine health check ups for young people are conducted once or twice a year. These do not depend upon the individual being unwell.

## Conclusion and Discussion

In general, this study showed that alcohol consumption (and tobacco smoking) were much less commonplace among Vietnamese young people than amongst young people in some other countries, such as those in Europe.^26^ But when we look at the gender and age factors, it was found that the percentage of alcohol drinkers and smokers is very high amongst adolescents of 18–19 age (29.5%), particular among the oldest ones (52%). Moreover, none of the Vietnamese girls surveyed reported that they smoked tobacco. This striking finding contrasts with the situation in some counties such as in Europe where girls are more likely to smoke (and in some cases to engage in ‘heavy episodic’ or ‘binge’ drinking) than boys.^26^–^28^ In fact, the levels of youthful alcohol use reported in this study were lower than those noted by earlier research in Vietnam.^29^ It should be noted that the present study related only to North Vietnam. It is possible that alcohol consumption is heavier in other parts of the country.

Drinking rates for all ages of sub populations remain different, but these rates are increasing in older groups and particularly amongst male populations, so alcohol use in young people is as much a public health problem as in adults.^30^ As in many other south and east Asian countries, it is common for groups of men to go out socially at night and drink alcohol.^31^ It is also a reminder that an increase in alcohol consumption also exerts a considerable economic burden worldwide.^32^ At the same time, compared to other Asian and Western countries, drinking rates in Vietnam still remain relatively low, but the prevalence of alcohol consumption among older male young people and currently drinking in this study is a little higher than other studies.^33^ In Vietnam, drinking is a social activity and occurs at family events such as religious memorials in honour of parents or grandparents who have died (cung gio cha me ong ba), or at parties, wedding days and birthdays. ‘Special occasions’ are increasing with economic development. Young people regularly take part in these events. Most males are encouraged to drink by others. Some use the expression: “A man without alcohol is like a flag without wind” (dan ong khong ruou nhu co khong phong). Several studies in Asia have noted that there has been rapid industrialization over the past three decades and South Korea.^34^,^35^ This change has resulted in greater prosperity. A study, conducted in northeastern India amongst 13–15-year old school students, concluded that the prevalence of smoking was 8.5 per cent-19.6 per cent amongst boys and 2.9–7.7 per cent amongst girls. The latter has been accompanied by increased production of alcoholic beverages and increased regular drinking, particularly beer and wine.^36^

Vietnam has no minimum legal age for purchasing alcohol and it is very easy for young people to get alcohol anywhere that it is on sale. Initiation into alcohol consumption occurred at an early age, even before the age of 18, particularly amongst males. The findings of the present study are a reminder that young people who begin drinking before they reach the age of 15 are significantly more likely to become heavy or dependent drinkers than those who begin drinking when they are older.^22^,^37^,^38^ With these economic and social changes, young people have had greater access to recreational activities and social meeting places such as cafes, small restaurants, karaoke, and bars.^33^ The leisure activities of young people include watching TV and playing video games. The latter often include acts of violence while under the influence of alcohol. advertising funded by the beer and wine producers.^39^ While Vietnam is still among the poorest countries in Asia, young people have more opportunities and threats in health.^40^ In the current research, the relationship between alcohol use and relevant factors suggests a link between alcohol use and risk behavior reduction prevention education and gender.^41^ As the country continues to undergo social and economic changes, it is essential to increase awareness of behaviours of alcohol use and its associated risks.^42^ Due note should be taken of both the short and long-term consequences of alcohol consumption among young people and the general population.

## Recommendations

This study showed that many both girls and boys were drinking. The young people surveyed had started to drink very early. Alcohol consumption was also related to risk taking, leisure activities and failure to access to health care services. Longitudinal research on alcohol use amongst Vietnamese young population should be conducted in Vietnam. This could monitor the trends of alcohol use and its related consequences. This could serve to guider the development of evidence-based policies to prevent, manage and minimize alcohol-related problems in Vietnam. As similar research conducted in other Asian countries such as Thailand and India has benefited prevention programmes.^43^^,44^

It is also recommended that alcohol harm reduction policies should be implemented and integrated into measures to reduce levels of other health problems such as HIV/AIDS and non communicable diseases. Such policies should ideally be evidence-based and evaluated.

## Figures and Tables

**Table 1:** Sample Design

Site	Stage		
	1st	2nd	3rd
	Province/city	Commune	Households
Urban (Ha Noi)	1	4	120
Rural (Ha Nam)	1	4	120
Mountain (Thai Nguyen)	1	4	120
Total	3	12	1 440

**Table 2:** Demographic characteristics of the sample

Variable	Overall (n = 1408)	Male (n = 673)	Female (n = 735)	OR	p
Age groups					
10–14 age	729 (51.8%)	358(53.2%)	371(50.5%)	1.12	0.3078
15–18 age)	679(48.2%)	315(46.8%)	364(49.5%)	0.90	0.3078
In-school	1322 (94%)	635 (94.3%)	687(93.5%)	1.01	0.902
Primary	312(24%)	153(24%)	159(23%)	1.05	0.6843
Secondary	596(45%)	289(46%)	307(45%)	1.03	0.7633
High school	400(30%)	187(29%)	213(31%)	0.93	0.5384
College	14(1%)	6(1%)	8(1%)	0.81	0.6967
Sites					
Urban	491(34.9%)	223(33.1%)	268(37%)	0.86	0.1906
Rural	477(33.9%)	236(35.1%)	241(33%)	1.11	0.3670
Mountain	440(31.3%)	214(31.8%)	226(31%)	1.05	0.6712

**Table 3:** Alcohol consumption and demographic factors

Characteristics	Overall n (%)	Male n (%)	Female n (%)	OR	p
Age					
10–14	67 (9.2)	38 (10.6)	29 (7.8)	1.4	0.191
15–19	166 (24.4)	117 (37.1)	49 (13.5)	4.4	0.000
Out-school	17 (19.8)	13 (34.2)	4 (8.3)	4.1	0.014
In-school	166 (12.5)	142 (22.3)	74 (10.8)	2.1	0.000
Primary	24 (7.7)	14 (9.2)	10 (6.3)	1.4	0.350
Secondary	75 (12.6)	49 (16.9)	26 (8.5)	2.0	0.005
High school	112 (28)	76 (40.6)	36 (16.9)	2.4	0.000
College/University	5 (36)	3 (50)	2 (25)	2.0	0.509
Areas					
Urban	65 (13.2)	41 (17.6)	24 (8.9)	2.05	0.007
Rural	70 (14.7)	45 (19.1)	25(10.4)	1.84	0.02
Mountain	98 (22.3)	69 (32.2)	29 (12.8)	2.51	0.000

**Table 4:** Television and, computer and computer game use

Characteristics	Overall n (%)	Male n (%)	Female n (%)	OR	p
Do nothing	141(10)	66 (9.8)	75 (10.2)	0.96	0.804
Less than 1 hour	123 (8.7)	56 (8.3)	67 (9.1)	0.90	0.597
1–2 hours	642 (45.6)	316 (46.9)	326 (44.3)	1.11	0.327
3–4 hours	411 (29.1)	189 (28)	222 (30.2)	0.89	0.335
5 or more hours	91 (6.5)	46 (6.8)	45 (6.1)	0.90	0.597

**Table 5:** Helmet Use amongst bicycle and motorbicycle riders

Variable	Overall n (%)	Male n (%)	Female n (%)	OR	P
Use bicycle/motorbicycle	1210 (86)	572 (85)	665 (70)	0.60	0.001
Always use	861 (61.2)	407 (60.5)	454 (61.8)	0.95	0.618
Sometimes	349 (24.8)	165 (24.5)	184 (25.0)	0.97	0.822
Never use	198 (14.1)	101 (15.0)	97 (13.2)	1.16	0.329
Wear helmet	268 (19.5)	134 (20.3)	134 (18.7)	1.12	0.422
Always	55 (6.4)	28 (4.2)	27(3.7)	1.14	0.637
Sometimes	213(15.1)	106(15.8)	107(14.6)	1.10	0.532
Never	1104(78.4)	523(77.7)	581(79.0)	0.92	0.542
